# Research trends on spinal muscular atrophy from 1995 to 2023: A bibliometric analysis

**DOI:** 10.1097/MD.0000000000041801

**Published:** 2025-03-28

**Authors:** Hao Yu, Cuijie Wei, Dan Sun, Li Zhang, Yanyan Xia, Wenhua Zhu

**Affiliations:** a Department of Medical Genetics and Center for Rare Diseases, Second Affiliated Hospital, Zhejiang University School of Medicine and Zhejiang Key Laboratory of Rare Diseases for Precision Medicine and Clinical Translation, Hangzhou, China; b Department of Pediatrics, Peking University First Hospital, Beijing, China; c Department of Neurology, Wuhan Children’ s Hospital Affiliated to Tongji Medical College, Huazhong University of Science and Technology, Wuhan, China; d Biogen Biotechnology (Shanghai) Co., Ltd, Shanghai, China; e Department of Neurology, Huashan Hospital of Fudan University, Shanghai, China.

**Keywords:** bibliometric analysis, neurodegenerative disease, SMN1, SMN2, spinal muscular atrophy

## Abstract

**Background:**

Spinal muscular atrophy (SMA) is a neuromuscular disease characterized by progressive muscle weakness due to motor neuron degeneration. The discovery of the survival motor neuron 1 (*SMN1*) gene in 1995 revolutionized SMA research, leading to significant therapeutic advancements. This bibliometric analysis aimed to explore global trends in SMA research and therapy, with a particular focus on China.

**Methods:**

A comprehensive database search identified 4506 relevant publications (3812 articles, 694 reviews) published between 1995 and 2023. Bibliometric tools were used to analyze publication trends, collaborations, and research topics.

**Results:**

SMA research has experienced substantial growth, with the United States leading in publications followed by the United Kingdom and Germany. China has shown increasing engagement in this field. Key research areas include genetic and molecular mechanisms, survival motor neuron gene therapy, antisense oligonucleotides, and muscle strength-promoting factors. Chinese researchers have contributed significantly to these areas, with a higher reporting frequency of SMA-related topics compared to other countries.

**Conclusion:**

This bibliometric analysis provides a comprehensive overview of global SMA research, highlighting significant advancements, and identifying future directions. The findings offer valuable insights for researchers, clinicians, and policymakers in China to ensure alignment with global medical advancements and improve the lives of individuals affected by SMA.

**Key points**

- This is the first comprehensive bibliometric analysis of spinal muscular atrophy research over 28 years.
- Overlapping and nonoverlapping research domains between other countries and China were identified.
- VOSviewer, bibliometric online tools, and Bibliometrix in R software were used for analysis.
- Key global contributors, journals, institutions, and collaborative scientists were identified, including those in China.
- Central interests in spinal muscular atrophy research and genetic focus in Chinese research are highlighted but limited by database and language constraints.

## 1. Introduction

Spinal muscular atrophy (SMA) is a devastating autosomal-recessive hereditary disorder that is pathologically characterized by wasting or irreversible degeneration of motor neurons, leading to muscular atrophy and loss of skeletal muscle movement.^[1]^ The incidence of SMA is ≈1 in 10,000 live births.^[2–5]^ The clinical stratification of SMA is based on the age of symptomatic manifestations and the degree of motor function deficits.^[6,7]^

The majority of patients with SMA (>90%) harbor a homozygous mutation in the survival motor neuron 1 (*SMN1*) gene, located at the 5q13.2 telomeric chromosomal locus of the human genome. A subset of these patients exhibit intragenic mutations that cause progressive motor neuron degeneration, manifesting as the SMA phenotype.^[1,8,9]^ Notably, multiple copies of *SMN2*, a centromeric homologue of *SMN1*, can exist in the same gene cluster and encode a protein that is 10 to 20% of the length of the functional SMN protein, thereby introducing a considerable degree of heterogeneity in disease phenotypes.^[10–13]^ The *SMN1* and *SMN2* genes differ only in 5 single nucleotide positions.^[1,14]^ However, only the c.840C > T mutation results in the skipping of exon 7 by converting the sequence to an exonic splice silencer,^[15,16]^ and the production of a highly unstable truncated version of the pathological SMN protein.^[17,18]^

The standard of care for patients with SMA involves interdisciplinary therapeutic interventions. However, a large degree of variability has been observed in strategic implementation across different patient populations.^[19]^ Recently, an updated version of the SMA standard of care has been developed.^[20]^ In addition to the updated SMA treatment guidelines, 3 therapeutic approaches have also taken the front line in SMA treatment: nusinersen, an antisense oligonucleotide-based therapy that can restore the normal splicing and function of *SMN*2 transcripts; gene therapy using onasemnogene abeparvovec, which produces a functional full-length copy of the human *SMN1* gene; and the small-molecule SMN2 splicing modifier risdiplam, which enhances U1 small nuclear ribonucleoprotein binding at exon 7, stabilizing the SMN2 pre-mRNA and U1 small nuclear ribonucleoprotein complex.^[21–25]^ To date, these 3 main therapeutic options can significantly prevent the worsening of SMA symptoms by increasing the level of SMN protein, but they are not curative treatments.^[26]^ Considering the wide range of phenotypic variability in SMA and the multiple failed clinical trials, it is essential to analyze the basic research and clinical trial data to pinpoint key disease modifiers or networks and design more effective therapies.

With the ongoing evolution of SMA treatment, it is crucial to have a comprehensive understanding and in-depth analysis of the status of and trends in SMA research. Moreover, statistical and visualization analyses using Bibliometrix in the R platform (R-Bibliometrix), CiteSpace, and VOSviewer can detail the scientific contributions made by different researchers, institutions/universities, countries, and journals to SMA disease research.^[27–31]^

In this analysis, we aimed to explore intractable roadblocks in SMA research and clinical development; highlight research hotspots and trends, especially in the development of novel therapeutic approaches over the past decades and; provide insights into future research and uncover disease-modifying factors in SMA by constructing knowledge graphs from scientific publications indexed in the Web of Science Core Collection and SCI-Expanded (WOS-SCIE) database from 1995 to 2023; this period encompasses the first report of the first report of the homozygous *SMN1* gene deletion mutation in 1995 to the present.^[1]^ Therefore, this analysis can provide the whole picture of SMA, and different status and trends between China and global.

## 2. Methods

### 2.1. Data sources and search strategy

Data analysis was performed using the open-source tools R-Bibliometrix (https://www.bibliometrix.org/home/) and VOSviewer (https://www.vosviewer.com/download), and another online analysis tool bibliometric. After adjusting and specifying the search strategy in the Medline target database, we used the following keyword strategy to search the WOS-SCIE database: (TI=(“spinal muscular atrophy”) OR AK=(“spinal muscular atrophy”) OR AB=(“spinal muscular atrophy”)) OR (TI=(“spinal muscular atrophies”) OR AK=(“spinal muscular atrophies”) OR AB=(“spinal muscular atrophies”)) OR (TI=(“spinal muscle atrophy”) OR AK=(“spinal muscle atrophy”) OR AB=(“spinal muscle atrophy”)) OR (TI=(“Werdnig Hoffman”) OR AK=(“Werdnig Hoffman”) OR AB=(“Werdnig Hoffman”)) OR (TI=(“Kugelberg Welander”) OR AK=(“Kugelberg Welander”) OR AB=(“Kugelberg Welander”)). The publication period of the retrieved articles ranged from January 1, 1995, to April 30, 2023. There were no ethical issues in obtaining the required data, and the requirement for ethical approval was waived.

### 2.2. Data collection and analytical methods

The open-source tools R-Bibliometrix and VOSviewer and another online analysis tool bibliometric were used to analyze and extract feature information of the articles from the WOS-SCIE database, including publication date, country, institution, language, research field, the number of publications by authors, and the number of publications by journals. The country, institution, and author were those mentioned in the publications. In the analysis of SMA-related publications by country, publications originating from Taiwan, Hong Kong, and Macau were included with those from mainland China; similarly, those from England, Scotland, Wales, and Northern Ireland were considered together as publications originating from the United Kingdom. R-Bibliometrix can quantitatively analyze relevant research articles in scientometrics and bibliometrics using the R language. VOSviewer and CiteSpace are used to construct and visualize bibliometric networks using a wide range of parameters.

## 3. Results

### 3.1. Overall development trajectory of SMA research

**Figure 1.**
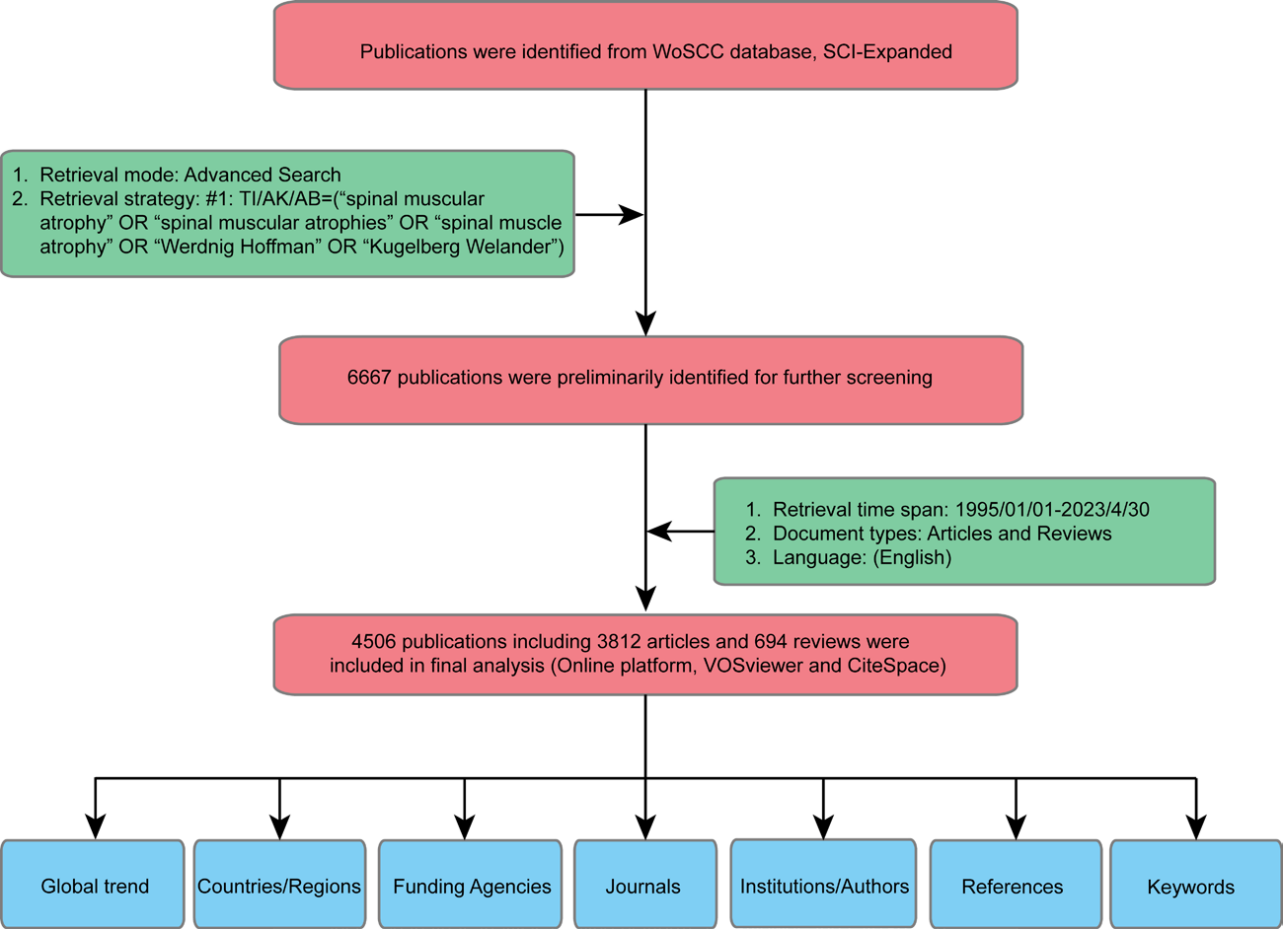
Schematic illustration of the search strategy for relevant articles in the Web of Science Core Collection database and subsequent selection processes.

**Figure 2.**
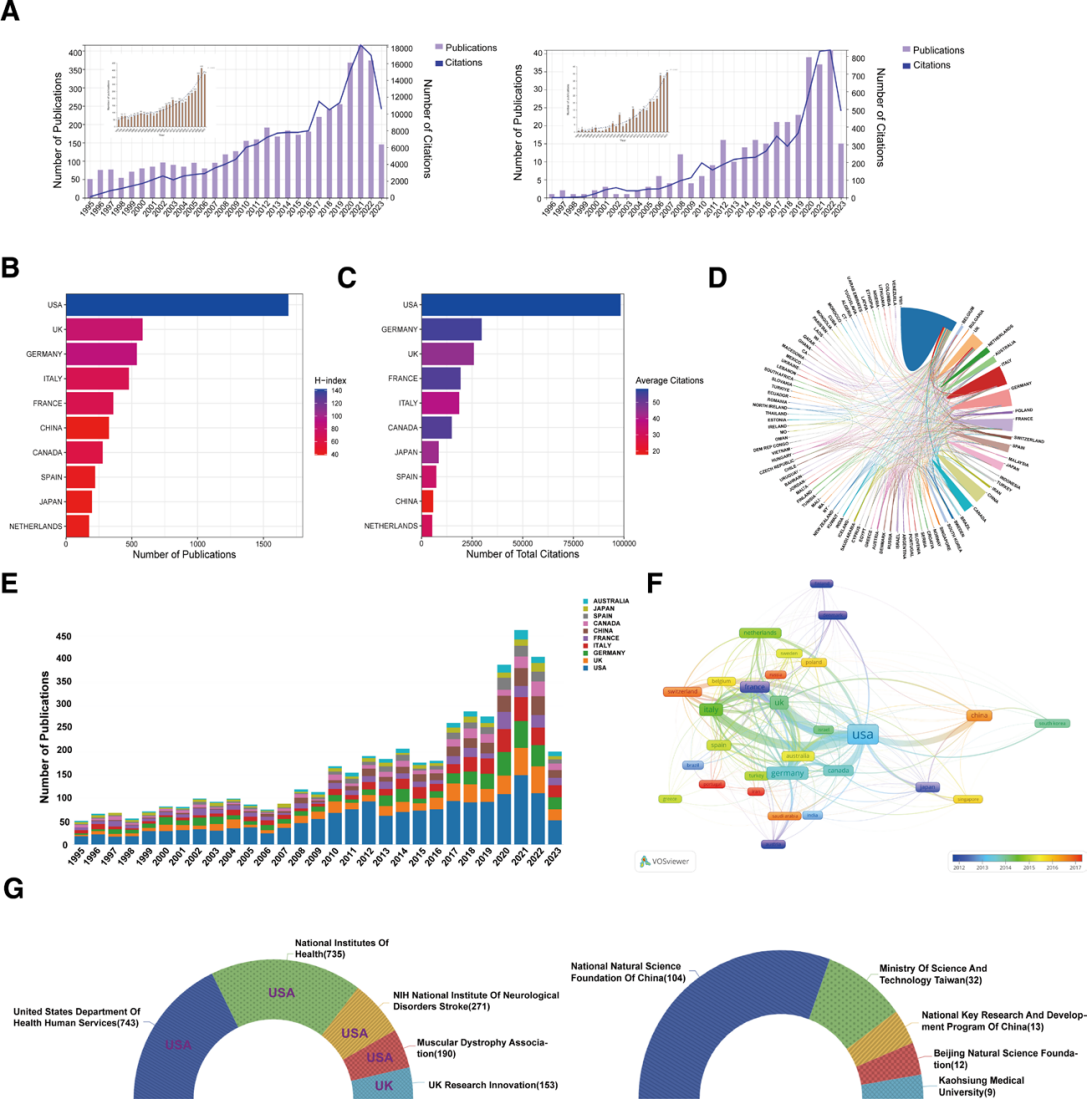
(A) Distribution patterns of annual numbers of SMA-related publications and corresponding citation counts globally (left) and in China (right). (B) Bar diagram showing the distribution of publication numbers from the top 10 countries. (C) Bar diagram showing the citation counts of the top 10 countries. (D) Visualization of global collaboration networks. (E) Distribution pattern of the number of publications by the top 10 countries each year. (F) Bibliometric country collaboration map visualization of the top 10 countries. Data analysis range: 4506 articles published in the WOS-SCIE database from 1995 to 2023; analysis tools: R-Bibliometrix, VOSviewer, WOS, Excel. Note: A combination of the H-index and average citation number was chosen as the indicator to evaluate contributions. The H-index was calculated by counting the number of publications for which an author has been cited by other authors at least that same number of times. A publication was attributed to multiple countries whether its authors were affiliated with institutions from different countries. (G) Half-donut pie chart visualization of the top 5 funding sources in all countries (left) and in China (right) for SMA-related research projects. SMA=spinal muscular atrophy, WOS-SCIE=Web of Science Core Collection and SCI-Expanded.

The pattern of scientific development in a designated field can be illustrated by assessing the change in publication frequency per year in peer-reviewed journals. Figure 1 shows the relevant publication retrieval and selection processes. Figure 2A shows the annual output trajectory of publications from 1995 to 2023 in SMA-related research areas. Over the past 28 years, a total of 6667 published articles related to SMA have been indexed in WOS-SCIE worldwide. Overall, 4506 publications including 3812 articles and 694 reviews were screened for the final analysis. Analysis of the global distribution of publications in each year revealed a significant increase from 51 publications in 1995 to 374 publications in 2022, with a peak of 416 articles in 2021. The cutoff for 2023 was April 30, 2023, and 145 articles were indexed in WOS-SCIE in this period. In China, a peak in publication number was observed in 2022, with a count of 41; 15 publications were included in the WOS-SCIE for 2023, with a cutoff of April 30, 2023. Notably, the frequency of publications increased from 2020 to 2022 but did not exhibit the variations observed in previous years, indicating a growing and persistent interest in SMA research.

### 3.2. Analysis of SMA-related publications by country

According to the WOS-SCIE registry, a total of 90 countries and regions contributed to SMA research. Figure 2B shows the top 10 contributing countries according to the total number of research articles published to date. The United States is the largest contributor to the SMA field (37.51%) with 1690 publications, followed by the United Kingdom (581 articles, 12.89%) and Germany (538 articles, 11.94%). Other major contributors in descending order of publication ranking were Italy (478 articles), France (359 articles), China (326 articles), Canada (279 articles), Spain (221 articles), Japan (198 articles), and the Netherlands (175 articles). Notably, China contributed 7.23% (326 publications) of the total number of SMA-related articles. The United States had the greatest number of citations, followed by Germany and the United Kingdom (Fig. 2C). Analysis of collaborations between countries revealed that the United States had the greatest number of collaborative projects with other countries (Fig. 2D). Figure 2E displays the number of publications from the top 10 countries in each year from 1995 to 2023, indicating an increasing trend in SMA-related publication counts. We also analyzed the co-authorships among these countries as shown in Figure 2F.

### 3.3. Analysis of the top institutions contributing to SMA research

Next, we identified the top institutions, universities, and hospitals contributing to SMA research. First, we analyzed the data of global and Chinese health research organizations contributing to SMA-related studies. The United States Department of Health and Human Services (743), National Institutes of Health (735), National Institute of Neurological Disorders and Stroke (271), Muscular Dystrophy Association (190), and the United Kingdom Research Innovation (153) were the top 5 organizations funding SMA research projects globally (Fig. 2G), while the National Natural Science Foundation of China (104), Ministry of Science and Technology Taiwan (32), National Key Research and Development Program of China (13), Beijing Natural Science Foundation (12), and Kaohsiung Medical University (9) were the top 5 funding sources for SMA research in China.

**Figure 3.**
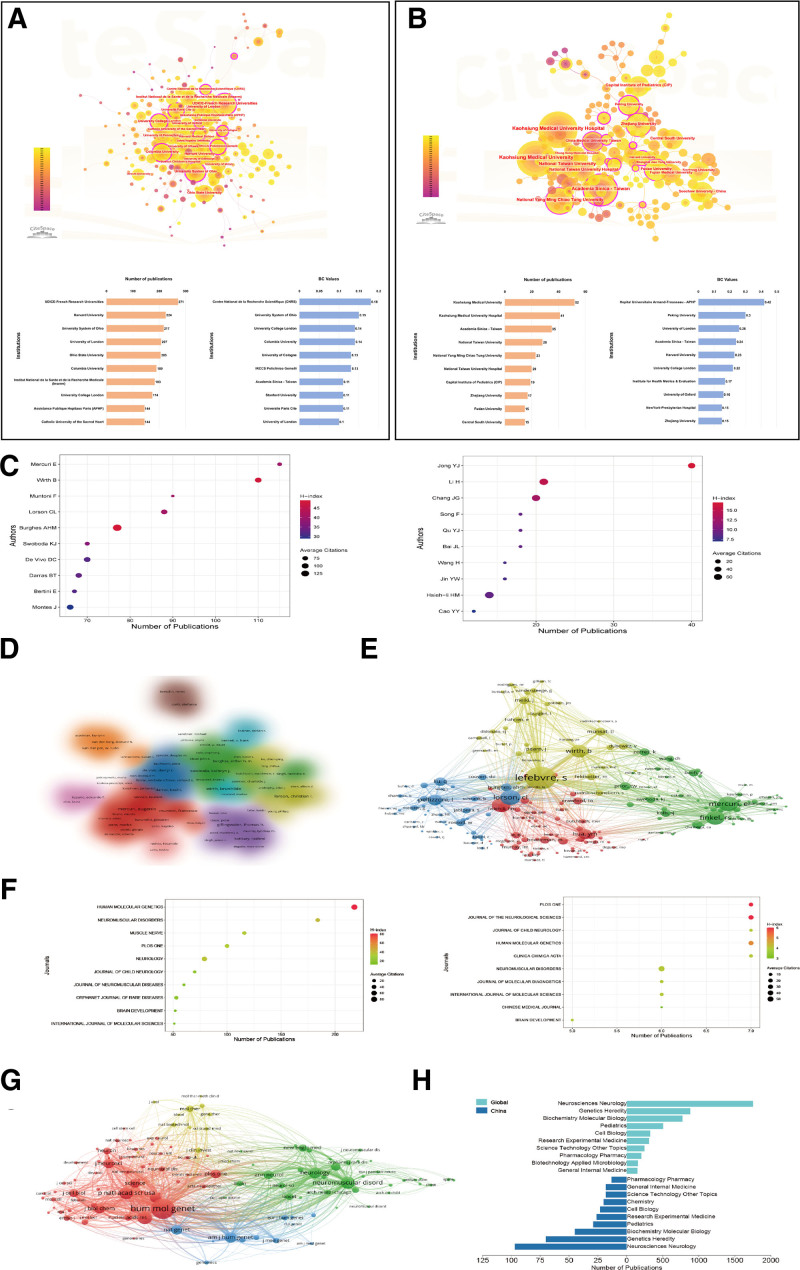
(A) Bibliometric visualization of the top 10 institutions, universities, and hospitals publishing SMA research globally. (Up) Visualization of clusters of highly contributing institutions globally. (Bottom left) Visualization of the top 10 institutions (in descending order) according to the number of SMA research publications globally. (Bottom right) Graphical representation of the top 10 institutions based on the betweenness centrality (BC) values globally. (B) Bibliometric visualization of the top 10 institutions, universities, and hospitals publishing SMA research in China. (Up) Visualization of clusters of highly contributing institutions in China. (Bottom left) Visualization of the top 10 institutions (in descending order) according to the number of SMA research publications in China. (Bottom right) Graphical representation of the top 10 institutions based on the BC values in China. Data analysis range: 4506 articles published in the WOS-SCIE database from 1995 to 2023; analysis tools: CiteSpace, R-Bibliometrix, Excel. (C) Bibliometric visualization of the top 10 authors contributing to the SMA field globally (left) and in China (right). (D) Cluster visualization of the authors of SMA publications. (E) Visualization of author collaborations in a node-cluster network. Data range: 323 papers published with Chinese affiliations in the WOS-SCIE database from 1995 to 2023; analysis tools: R-Bibliometrix, Excel. The institutional ranking in this study was determined by R-Bibliometrix, which had an automatic disambiguation option for institutions. (**F**) Visualization of journal rankings by the number of articles published by international authors (left) and Chinese authors (right). (G) Bibliometric node-cluster network visualization of relevant journals based on SMA-related topics. (H) Ranking of research directions of publications by international and Chinese authors. Data range: 323 papers published with Chinese affiliations in the WOS-SCIE database from 1995 to 2023; analysis tools: R-Bibliometrix, Excel. Note: The size of the dots represents the average number of citations, with larger dots indicating journals that have published articles with higher average citation counts. This principle is also used in the calculation of impact factors. The horizontal axes represent the number of publications and the number of citations. The color coding reflects the H-index. SMA=spinal muscular atrophy, WOS-SCIE=Web of Science Core Collection and SCI-Expanded.

The WOS-SCIE database indicates the involvement of >4000 institutions in SMA research and therapy globally (Fig. 3A). Among these, the UDICE-French Research University published the most studies (271 articles) from 1995 to 2023, followed by Harvard University (224 articles), the University System of Ohio (217 articles), the University of London (207 articles), Ohio State University (205 articles), Columbia University (189 articles), Institute National de la Sante et de la Research Medical (183 articles), University College London (174 articles), and Assistance Publique Hopitaux de Paris (144 articles), which were the top 10 contributors to the SMA field. Similarly, to identify the top-contributing Chinese institutions and universities, we analyzed the total number of SMA-linked publications from nationwide academic and research institutions in China (including Taiwan) from 1995 to 2023 (Fig. 3B). Kaohsiung Medical University ranked first with 52 publications, followed by Kaohsiung Medical University Hospital (41 articles) and Academia Sinica - Taiwan (35 articles).

### 3.4. Analysis of authors’ contributions

Next, we analyzed the authors’ contributions based on the number of publications on SMA from all countries and with Chinese affiliations in the past 28 years (Fig. 3C through 3E). At the international level, E. Mercuri had the greatest number of published articles (115), with an average of 62.76 citations per publication. B. Wirth ranked second, with 110 publications and an average number of citations of 91.34 per paper, and F. Muntoni was the third most published author, with 90 articles and an average number of citations of 56.52 per article. However, A.H.M. Burghes had the greatest average number of citations and the highest H-index in this group (Fig. 3C). Among Chinese authors, Y.J. Jong had the greatest number of published articles (40) with an average citation of 46.7 per paper. H. Li published 21 articles with an average citation of 73.67 per paper, followed by J.G. Chang, with 20 publications and 67.1 average citations per paper (Fig. 3C). In total, 107 authors who published more than 15 articles were included in the cluster analysis, and 12 clusters were identified, as shown in Figure 3D. Figure 3E displays a representation of the total number of citations for each author, with the size of the node representing the number of citations. The larger the node is, the greater the impact of the author.

### 3.5. Analysis of the journal distribution of SMA-related publications

**Figure 4.**
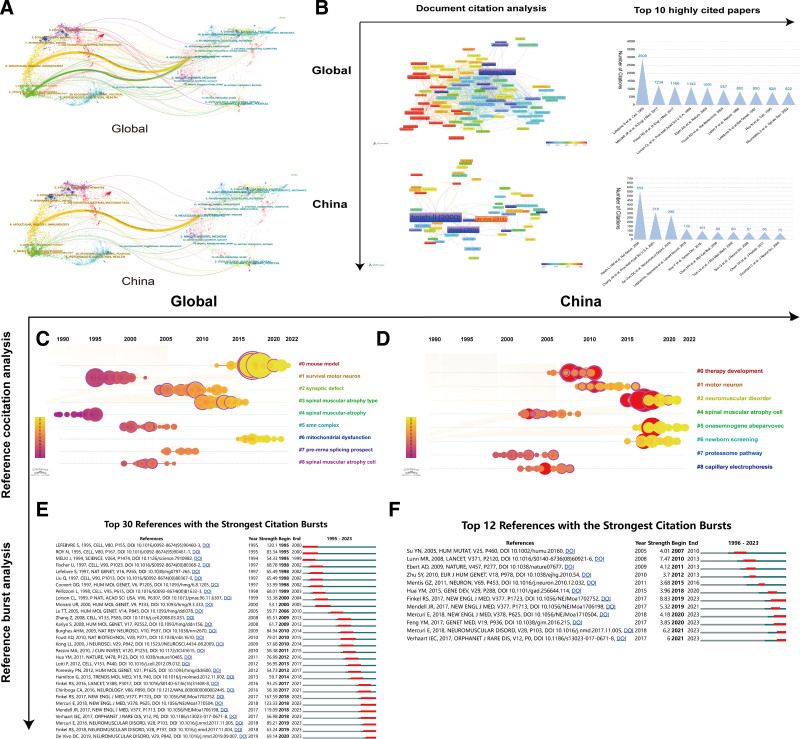
(A) Two-field plot of journals and their SMA-related topics published by international (up) and Chinese (bottom) authors. Data range: 323 papers published with Chinese affiliations in the WOS-SCIE database from 1995 to 2023; analysis tools: R-Bibliometrix, Excel. (B) Visualization of the author-journal collaboration network and top-cited SMA-related articles globally and in China. Data range: 4506 papers published in the WOS-SCIE database from 1995 to 2023; analysis tools: R-Bibliometrix, VOSviewer. (C through F) Bibliometric visualization of the reference co-citations (C and D) and reference burst (E and F) for global (C and E) and Chinese (D and F) authors. Data range: 323 published articles in the WOS-SCIE database from 1995 to 2023; analysis tools: R-Bibliometrix, Excel. SMA=spinal muscular atrophy, WOS-SCIE=Web of Science Core Collection and SCI-Expanded.

We the top 10 peer-reviewed journals that published SMA-related articles in the past 28 years. Globally, we found that Human Molecular Genetics published the greatest number of articles (218) with the highest H-index and a total of ~18,000 citations. In descending order, the other top journals included Neuromuscular Disorders (184 articles), Muscle & Nerve (116 articles), PLOS ONE (100 articles), Neurology (79 articles), Journal of Child Neurology (70 articles), Journal of Neuromuscular Diseases (60 articles), Brain & Development (52 articles), and International Journal of Molecular Sciences (51 articles; Fig. 3F). The top 10 journals that published articles on SMA written mostly by Chinese authors were PLOS ONE, Journal of the Neurological Sciences, Journal of Child Neurology, Human Molecular Genetics, and Clinica Chimica Acta, which were all jointly ranked first, with 7 articles each, followed by Neuromuscular Diseases (6 articles), Journal of Molecular Diagnostic (6 articles), International Journal of Molecular Sciences (6 articles), Chinese Medical Journal (6 articles), and Brain & Development (5 articles; Fig. 3F). Furthermore, bibliometric network visualization of SMA-related articles published in journals consistently indicated that Human Molecular Genetics was the top-ranking journal (Fig. 3G). Figure 3H displays the ranking of the top 10 research directions in the SMA field by global investigators (upper half) and Chinese authors (lower half). Two-field plots show the correlative distributions of journals and topics of published SMA articles by global and Chinese authors (Fig. 4A).

### 3.6. Analysis of author-journal collaboration networks and top-cited publications

We next analyzed the collaborations of authors and top-cited publications globally and in China. Based on the number of citations of the SCI publications, S. Lefebvre and H.M. Hsieh-Li ranked highest in the collaboration network globally and in China (Fig. 4B), respectively. The most cited article was published by S. Lefebvre et al in 1995 in Cell Journal, with 2908 total citations. The second-ranked article was written by J.R. Mendell et al, who published in the New England Journal of Medicine in 2017, which received a total of 1234 citations. The third-ranked article was written by R.S. Finkel et al and published in New England Journal of Medicine in 2017, with 1166 citations. In China, the article published in Nature Genetics by H.M. Hsieh-Li et al had the highest number of citations at 559. This article described the pathological effect of a homozygous *SMN1* mutation in a mouse model of SMA. The second-ranked article was written by J.G. Chang et al and published in Proceedings of the National Academy of Sciences of the United States of America in 2001, with 318 citations; this article reported the therapeutic potential of sodium butyrate in a rodent model of SMA. The third most cited paper was written by D.C. De Vivo et al and published in the Journal of Neuromuscular Disorders in 2019, with 286 citations; the article reported the clinical efficacy of nusinersen in pediatric patients under presymptomatic conditions.

### 3.7. Analysis of reference co-citations and reference bursts

The reference co-citation analysis revealed that mouse model, survival motor neuron, synaptic defect, SMA type, SMA complex, mitochondrial dysfunction, pre-mRNA splicing prospect, and SMA cell were the top 8 research directions globally (Fig. 4C and 4E). We also identified the top 30 references cited by international authors with the strongest citation bursts; the study published by S. Lefebvre in Cell in 1995 ranked first, with a strength score of 120.1. In China, the research fields with the most co-citations were therapy development, motor neuron, neuromuscular disorder, SMA cell, onasemnogene abeparvovec, newborn screening, proteasome pathway, and capillary electrophoresis (Fig. 4D and 4F); the article published by Y.N. Su in Human Mutation in 2005 had the strongest citation burst, with a strength index of 4.01.

### 3.8. Analysis of SMA-related keyword trends

**Figure 5.**
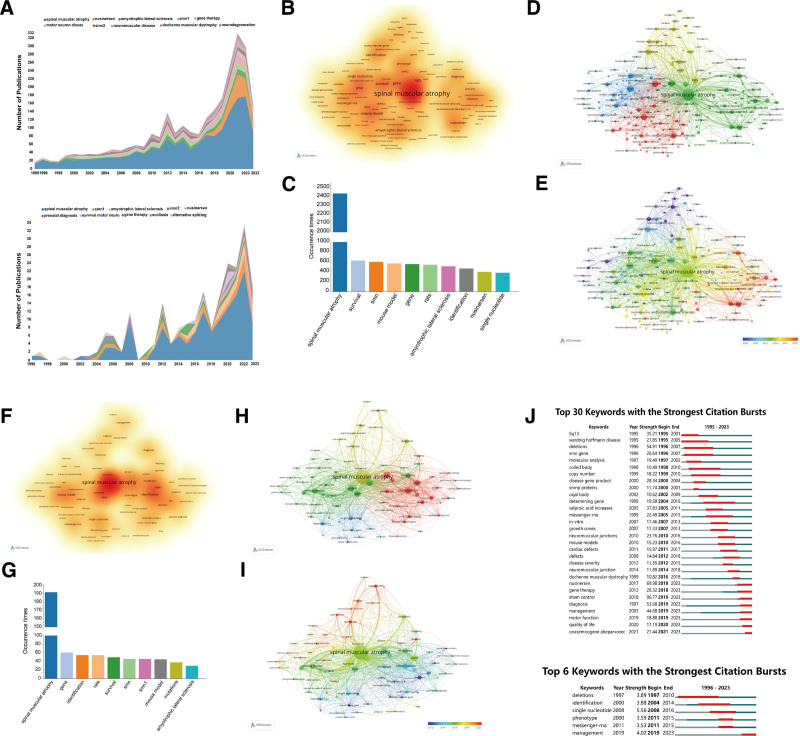
(A) Bibliometric visualization of the SMA-related keyword frequencies in international (up) and Chinese institution-associated (bottom) publications. Data range: 323 papers published with Chinese affiliations in the WOS-SCIE database from 1995 to 2023; analysis tools: R-Bibliometrix. (B through E)Visualization of the global co-occurrence analysis. (B) Density view of keywords. (C) Co-occurrences of major search items. (D) Network visualization of relevant keywords. (E) Visualization of the overlay network map. Data range: 323 papers published with Chinese affiliations in the WOS-SCIE database from 1995 to 2023; analysis tools: R-Bibliometrix, VOSviewer. (F through I)Visualization of the co-occurrence analysis in Chinese publications. (F) Density view of keywords. (G) Co-occurrences of major search items. (H) Network visualization of relevant keywords. (I) Visualization of the overlay network map. Data range: 323 papers published with Chinese affiliations in the WOS-SCIE database from 1995 to 2023; analysis tools: R-Bibliometrix, VOSviewer. (J) Bibliometric visualization of the keywords with the strongest citation bursts based on international (up) and China-affiliated (bottom) publications. Data range: 323 papers published with Chinese affiliations in the WOS-SCIE database from 1995 to 2023; analysis tools: R-Bibliometrix. SMA=spinal muscular atrophy, WOS-SCIE=Web of Science Core Collection and SCI-Expanded.

Analysis of the most frequently searched or utilized keywords can indicate trends in scientific research and therapy for SMA since 1995. By analyzing the yearly frequency of SMA-related keyword usage in relevant international publications, we found increasing trends for the following keywords: SMA, nusinersen, ALS, *SMN1*, gene therapy, motor neuron disease, *SMN2*, neuromuscular disease, Duchenne muscular dystrophy, and neurodegeneration (Fig. 5A). Among Chinese publications, the most frequently used keywords were SMA, *SMN1*, ALS, *SMN2*, nusinersen, prenatal diagnosis, survival motor neuron, gene therapy, scoliosis, and alternative splicing (Fig. 5A). Overall, there has been a steadily increasing trend in the number of publications on SMA over the past 28 years when searched using these keywords.

### 3.9. Analysis of co-occurrences of SMA-related keywords

A co-occurrence analysis can identify relationships between different concepts and terms within a body of literature, which can be instrumental in advancing knowledge in that field. We conducted co-occurrence analyses to understand ongoing research trends in publications on SMA. Here, we analyzed 195 keywords with co-occurrence frequencies >30 in international publications and determined the top 10 global search terms, namely, SMA (2440 times), survival (629 times), SMN (605 times), mouse model (572 times), gene (559 times), rates (546 times), ALS (515 times), identification (470 times), nusinersen (402 times), and single nucleotide (385 times; Fig. 5B and 5C). Since “spinal muscular atrophy” was the central term of the retrieval strategy and any related research works included those keywords, it appeared to be the highest search item both globally and in Chinese publications. The network visualization map (Fig. 5D) revealed 4 major clusters of the most used keywords, and the overlay view map (Fig. 5E) displayed the trend of those keywords in a time-dependent manner. In the Chinese publications, the top 10 keywords were SMA (192 times), gene (61 times), identification (55 times), rats (55 times), survival (50 times), SMN (46 times), SMN1 (46 times), mouse model (45 times), mutations (38 times), ALS (30 times; Fig. 5F and 5G). Similarly, the network visualization map (Fig. 5H) and overlay view map showed that SMA was the most frequent co-occurring search term (Fig. 5I).

### 3.10. Analysis of keywords with the strongest citation bursts

Finally, we analyzed the strength of the citation bursts using all the keywords from internationally published articles and published articles with Chinese affiliations. First, we identified the top 30 keywords with significantly strong citation bursts in international publications; the top 5 keywords were 5q13, Werdnig Hoffmann disease, deletions, SMN gene, and molecular analysis, with corresponding strength indices of 35.21, 27.85, 54.91, 26.64, and 19.49, respectively (Fig. 5J). Similarly, the top 5 keywords in China-affiliated publications were deletions, identification, single nucleotide, phenotype, mRNA, and management, with strength indices of 3.89, 3.88, 5.56, 3.59, and 3.53, respectively (Fig. 5J). Notably, in Chinese publications, only 6 keywords (mostly identified in international publications) were detected.

## 4. Discussion

We conducted a bibliometric analysis on 4506 publications screened from the WOS-SCIE database using R-Bibliometrix and VOSviewer to construct visualization maps and tables to vividly display the research hotspots, status, and trends in recent clinical and basic research on SMA. We found that there has been increased interest in SMA globally since 2017, with a steady increase since 2019 and the highest number of SMA-related publications (n = 416) in 2021. Although the United States had the greatest global contribution, with 1690 publications on SMA, the growing interest of Chinese scientists was noticeable, with 7.23% of the total number of included articles, which was close to the contributions of France but more than those of Spain and Japan, suggesting that Chinese scholars have been increasingly involved in finding a curative personalized therapy for this fatal disease. This trend was consistently reflected in the extensive collaboration and increased number of publications by Chinese scholars. One major reason for this trend could be the inclusion of nusinersen, which is used to treat the *SMN2* gene mutation on chromosome 5q11.2-q13.3,^[1]^ on the national reimbursement list of life-saving drugs in China in 2022.

Given that the SMA disease burden is continuously increasing worldwide, including in the United States and China,^[32,33]^ national funding bodies are beginning to invest more in SMA diagnosis and drug discovery research. We observed that the US Department of Health and Human Resources and United Kingdom Research Innovation were the 2 major funding bodies in the United States and United Kingdom, respectively, while the National Natural Science Foundation of China was the leading funding organization among Chinese academic and industrial research centers promoting SMA research. Notably, collaborative research has also increased among international and Chinese academics in recent years. In China, Kaohsiung Medical University and Academia Sinica, Taiwan, are the 2 top-tier institutions for researching SMA, ranging from basic mechanisms to clinical studies. In addition to these institutions or universities, investigators from other institutes also contributed significantly to peer-reviewed international journals, particularly E. Mercuri (Italy) and B. Wirth (Germany), who were the first and second highest published authors on SMA, respectively. In China, the author with the greatest number of publication, Y.J. Jong, was associated with the top-tier institution, Kaohsiung Medical University Hospital, in the field of SMA.

Furthermore, an analysis of journals that published numerous SMA-related articles indicates that Human Molecular Genetics was ranked first, with 218 articles through April 30, 2023. Chinese authors published mostly in PLOS ONE, the Journal of Neurological Sciences, the Journal of Child Neurology, Human Molecular Genetics, and Clinica Chimica Acta. Gene therapy and alternative splicing have gained the most attention among Chinese researchers because of the increasing incidence of SMA in newborns in China.^[34–36]^ Similarly, the 5 keywords with the most citations were 5q13, Werdnig Hoffmann disease (SMA type I),^[37]^ deletions, SMN gene, and molecular analysis, suggesting the importance of SMA-related topics in the contemporary clinical field of neurodegenerative diseases. During the analysis period, there was increased interest in deciphering the genetics of SMA among Chinese researchers, which was reflected by the top 3 citation bursts. Notably, there was a difference in research hotspots between China and other countries, indicating that the research perspectives vary among SMA researchers. However, we hope that these 2 directions will converge at 1 point, providing holistic knowledge to develop efficient personalized therapy for individuals with SMA.

Aligning these findings with our analysis, it is evident that there is a significant search frequency associated with certain terms, including SMA, gene therapy, motor neuron disease, etc. We can visualize the trending topics on SMA in the cluster analysis, which have been predominantly therapeutics and their applications over the past few years. Thematic mapping of research topics and trends indicates that Chinese scholars have focused on understanding disease mechanisms, phenotype determination, and patient survival. In contrast, the global trend seems to be directed towards investigating potential links between SMA and amyotrophic lateral sclerosis (ALS) by identifying deletions, single nucleotides, phenotypes, mRNAs, etc.

Over the past 28 years, an exhaustive number of in-depth studies have been conducted in multiple research areas related to SMA and associated neurodegeneration. However, improvements in biomarker-based SMA diagnosis and disease phenotype-oriented personalized therapy are lacking. Therefore, in-depth longitudinal studies and broad-range randomized controlled trials are important to consolidate the present research findings.

Overall, this is the first bibliometric analysis of SMA research over the past 28 years, delineating various global areas of interest in SMA research. Bibliometric analysis provides a method to obtain a holistic overview of SMA research and clinical investigations, which might help Chinese health care practitioners develop future therapies to save the lives of children.

## 5. Strengths and limitations

To our knowledge, this is the first bibliometric analysis to comprehensively analyze multiple cohort studies and clinical trials on SMA published over the past 28 years. This analysis revealed overlapping and nonoverlapping research domains between international countries and China regarding SMA-related research. Using VOSviewer, the online analysis tool bibliometric and R-Bibliometrix, we constructed multiple knowledge maps and tables to illustrate the development trajectory of SMA research and management. We also identified countries, peer-reviewed journals, highly contributing academic institutions, and the most collaborative scientists worldwide, including China. The thematic map and subject trend analysis increased our understanding of the current trends in SMA research from both global and Chinese perspectives. Moreover, we found that different therapeutic approaches and the development of novel drug targets have been the central interest in SMA research globally, whereas Chinese researchers have focused more on deciphering the genetic factors that drive SMA onset in pediatric patients. In-depth knowledge of the detailed pathomechanism is likely to accelerate the discovery and application of more advanced small-molecule therapeutics. However, this analysis has certain limitations. First, almost all publications were retrieved from the WOS-SCIE database, while a very small portion of the total included articles were obtained from Medline, which might limit the generalizability of our findings. Second, we included only journal articles and reviews and not conference proceedings or book chapters. Third, we only collected English language publications from the database that had been published over the past 28 years using specific search strings but did not include other related articles in the field, which might have influenced the overall summary of the results.

In the future, more comprehensive studies, including multiple databases, and multiple literature types, are warranted to further validate and generalize our findings. Nevertheless, this bibliometric analysis provided new insights into recent advancements in SMA research and identified potential challenges that could create a roadblock for rapid progress.

## 6. Conclusion

Overall, our results elucidated recent trends in the field of SMA research over the past 28 years and provide a more in-depth understanding of the disease mechanisms and treatment responses of pediatric patients with different subtypes of SMA. First, we observed that the research interest in SMA has increased steadily in recent years, indicating that this is still an emerging field, and further comprehensive studies are needed to develop curative therapeutics for SMA. Second, the SMA research was relatively multidisciplinary, involving public health, neurology, nutrition, and psychology. Third, most articles were published by Western countries, particularly by researchers in the United States. Although scientific collaboration can be influenced by geographical differences, cultural barriers, and language, Chinese scholars have exhibited significant improvements in building a global collaborative network. Therefore, it is necessary to promote these initiatives by conducting international symposiums on SMA. Fourth, the analysis of topic trends in China indicates that the most recent (2022) clinical research interest has been centered on nusinersen and its therapeutic application in pediatric patients with SMA. In summary, our analysis offers a comprehensive view of SMA research to date, assisting researchers worldwide and in China in understanding the current publication status and key areas of focus. This analysis aimed to minimize redundant studies on already discussed topics and provide a clear research direction for Chinese researchers.

## Author contributions

**Conceptualization:** Hao Yu, Cuijie Wei, Wenhua Zhu.

**Formal analysis:** Hao Yu, Cuijie Wei, Dan Sun, Wenhua Zhu.

**Investigation:** Hao Yu, Li Zhang, Wenhua Zhu.

**Methodology:** Hao Yu, Li Zhang, Wenhua Zhu.

**Project administration:** Hao Yu, Li Zhang, Wenhua Zhu.

**Validation:** Hao Yu, Cuijie Wei, Dan Sun, Wenhua Zhu.

**Visualization:** Hao Yu, Cuijie Wei, Dan Sun, Yanyan Xia, Wenhua Zhu.

**Writing—original draft:** Hao Yu, Cuijie Wei, Dan Sun, Yanyan Xia, Wenhua Zhu.

**Writing—review & editing:** Hao Yu, Dan Sun, Yanyan Xia, Wenhua Zhu.

**Data curation:** Dan Sun, Wenhua Zhu.

**Supervision:** Yanyan Xia.

**Funding acquisition:** Wenhua Zhu.

**Resources:** Wenhua Zhu.

**Software:** Wenhua Zhu.

## References

[1] Lefebvre S Bürglen L Reboullet S. Identification and characterization of a spinal muscular atrophy-determining gene. Cell. 1995;80:155–65. 7813012 10.1016/0092-8674(95)90460-3

[2] Arkblad E Tulinius M Kroksmark AK Henricsson M Darin N. A population-based study of genotypic and phenotypic variability in children with spinal muscular atrophy. Acta Paediatr. 2009;98:865–72. 19154529 10.1111/j.1651-2227.2008.01201.x

[3] Jedrzejowska M Milewski M Zimowski J. Incidence of spinal muscular atrophy in Poland--more frequent than predicted? Neuroepidemiology. 2010;34:152–7. 20090376 10.1159/000275492

[4] Prior TW Snyder PJ Rink BD. Newborn and carrier screening for spinal muscular atrophy. Am J Med Genet A. 2010;152A:1608–16. 20578137 10.1002/ajmg.a.33474

[5] Sugarman EA Nagan N Zhu H. Pan-ethnic carrier screening and prenatal diagnosis for spinal muscular atrophy: clinical laboratory analysis of >72,400 specimens. Eur J Hum Genet. 2012;20:27–32. 21811307 10.1038/ejhg.2011.134 PMC3234503

[6] Finkel R Bertini E Muntoni F Mercuri E; ENMC SMA Workshop Study Group. 209th ENMC International Workshop: outcome measures and clinical trial readiness in spinal muscular atrophy 7–9 November 2014, Heemskerk, The Netherlands. Neuromuscul Disord. 2015;25:593–602. 26045156 10.1016/j.nmd.2015.04.009

[7] Lunn MR Wang CH. Spinal muscular atrophy. Lancet. 2008;371:2120–33. 18572081 10.1016/S0140-6736(08)60921-6

[8] Wirth B Herz M Wetter A. Quantitative analysis of survival motor neuron copies: identification of subtle SMN1 mutations in patients with spinal muscular atrophy, genotype-phenotype correlation, and implications for genetic counseling. Am J Hum Genet. 1999;64:1340–56. 10205265 10.1086/302369 PMC1377870

[9] Markowitz JA Singh P Darras BT. Spinal muscular atrophy: a clinical and research update. Pediatr Neurol. 2012;46:1–12. 22196485 10.1016/j.pediatrneurol.2011.09.001

[10] Gennarelli M Lucarelli M Capon F. Survival motor neuron gene transcript analysis in muscles from spinal muscular atrophy patients. Biochem Biophys Res Commun. 1995;213:342–8. 7639755 10.1006/bbrc.1995.2135

[11] Feldkötter M Schwarzer V Wirth R Wienker TF Wirth B. Quantitative analyses of SMN1 and SMN2 based on real-time lightCycler PCR: fast and highly reliable carrier testing and prediction of severity of spinal muscular atrophy. Am J Hum Genet. 2002;70:358–68. 11791208 10.1086/338627 PMC419987

[12] Wirth B Brichta L Schrank B. Mildly affected patients with spinal muscular atrophy are partially protected by an increased SMN2 copy number. Hum Genet. 2006;119:422–8. 16508748 10.1007/s00439-006-0156-7

[13] McAndrew PE Parsons DW Simard LR. Identification of proximal spinal muscular atrophy carriers and patients by analysis of SMNT and SMNC gene copy number. Am J Hum Genet. 1997;60:1411–22. 9199562 10.1086/515465 PMC1716150

[14] Costa-Roger M Blasco-Pérez L Cuscó I Tizzano EF. The importance of digging into the genetics of SMN genes in the therapeutic scenario of spinal muscular atrophy. Int J Mol Sci. 2021;22:9029. 34445733 10.3390/ijms22169029 PMC8396600

[15] Cartegni L Krainer AR. Disruption of an SF2/ASF-dependent exonic splicing enhancer in SMN2 causes spinal muscular atrophy in the absence of SMN1. Nat Genet. 2002;30:377–84. 11925564 10.1038/ng854

[16] Kashima T Manley JL. A negative element in SMN2 exon 7 inhibits splicing in spinal muscular atrophy. Nat Genet. 2003;34:460–3. 12833158 10.1038/ng1207

[17] Lorson CL Hahnen E Androphy EJ Wirth B. A single nucleotide in the SMN gene regulates splicing and is responsible for spinal muscular atrophy. Proc Natl Acad Sci USA. 1999;96:6307–11. 10339583 10.1073/pnas.96.11.6307 PMC26877

[18] Monani UR Lorson CL Parsons DW. A single nucleotide difference that alters splicing patterns distinguishes the SMA gene SMN1 from the copy gene SMN2. Hum Mol Genet. 1999;8:1177–83. 10369862 10.1093/hmg/8.7.1177

[19] Bladen CL Thompson R Jackson JM. Mapping the differences in care for 5,000 spinal muscular atrophy patients, a survey of 24 national registries in North America, Australasia and Europe. J Neurol. 2014;261:152–63. 24162038 10.1007/s00415-013-7154-1

[20] Mercuri E Finkel RS Muntoni F; SMA Care Group. Diagnosis and management of spinal muscular atrophy: part 1: recommendations for diagnosis, rehabilitation, orthopedic and nutritional care. Neuromuscul Disord. 2018;28:103–15. 29290580 10.1016/j.nmd.2017.11.005

[21] Chiriboga CA Swoboda KJ Darras BT. Results from a phase 1 study of nusinersen (ISIS-SMN(Rx)) in children with spinal muscular atrophy. Neurology. 2016;86:890–7. 26865511 10.1212/WNL.0000000000002445 PMC4782111

[22] Finkel RS Mercuri E Darras BT; ENDEAR Study Group. Nusinersen versus Sham control in infantile-onset spinal muscular atrophy. N Engl J Med. 2017;377:1723–32. 29091570 10.1056/NEJMoa1702752

[23] Poirier A Weetall M Heinig K. Risdiplam distributes and increases SMN protein in both the central nervous system and peripheral organs. Pharmacol Res Perspect. 2018;6:e00447. 30519476 10.1002/prp2.447 PMC6262736

[24] Mendell JR Al-Zaidy S Shell R. Single-dose gene-replacement therapy for spinal muscular atrophy. N Engl J Med. 2017;377:1713–22. 29091557 10.1056/NEJMoa1706198

[25] Hardcastle N Boulis NM Federici T. AAV gene delivery to the spinal cord: serotypes, methods, candidate diseases, and clinical trials. Expert Opin Biol Ther. 2018;18:293–307. 29249183 10.1080/14712598.2018.1416089

[26] Day JW Howell K Place A. Advances and limitations for the treatment of spinal muscular atrophy. BMC Pediatr. 2022;22:632. 36329412 10.1186/s12887-022-03671-x PMC9632131

[27] Brandt JS Hadaya O Schuster M Rosen T Sauer MV Ananth CV. A bibliometric analysis of top-cited journal articles in obstetrics and gynecology. JAMA Netw Open. 2019;2:e1918007. 31860106 10.1001/jamanetworkopen.2019.18007 PMC6991228

[28] Wilson M Sampson M Barrowman N Doja A. Bibliometric analysis of neurology articles published in general medicine journals. JAMA Netw Open. 2021;4:e215840. 33856477 10.1001/jamanetworkopen.2021.5840 PMC8050738

[29] He T Wu Z Zhang X. A Bibliometric analysis of research on the role of BDNF in depression and treatment. Biomolecules. 2022;12:1464. 36291673 10.3390/biom12101464 PMC9599058

[30] Chen C Hu Z Liu S Tseng H. Emerging trends in regenerative medicine: a scientometric analysis in CiteSpace. Expert Opin Biol Ther. 2012;12:593–608. 22443895 10.1517/14712598.2012.674507

[31] Tu JX Lin XT Ye HQ. Global research trends of artificial intelligence applied in esophageal carcinoma: a bibliometric analysis (2000-2022) via CiteSpace and VOSviewer. Front Oncol. 2022;12:972357. 36091151 10.3389/fonc.2022.972357 PMC9453500

[32] Mouchet J Roumpanis S Gaki E. Disease burden of spinal muscular atrophy: a comparative cohort study using insurance claims data in the USA. J Neuromuscul Dis. 2023;10:41–53. 36314213 10.3233/JND-210764 PMC9881018

[33] Li C Geng Y Zhu X. The prevalence of spinal muscular atrophy carrier in China: Evidences from epidemiological surveys. Medicine (Baltimore). 2020;99:e18975. 32000428 10.1097/MD.0000000000018975 PMC7004774

[34] Jia C Zhao D Li Y. Newborn screening and genomic analysis of duchenne muscular dystrophy in Henan, China. Clin Chim Acta. 2023;539:90–6. 36516925 10.1016/j.cca.2022.11.024

[35] Chan SHS Lo IFM Cherk SWW. Prevalence and characteristics of chinese patients with Duchenne and Becker muscular dystrophy: a territory wide collaborative study in Hong Kong. Child Neurol Open. 2015;2:2329048X15585345. 10.1177/2329048X15585345 PMC5417024 28503591

[36] Hu C Li X Shi Y. Comprehensive profile and natural history of pediatric patients with spinal muscular atrophy: a large retrospective study from China. Front Neurol. 2022;13:1038012. 36605788 10.3389/fneur.2022.1038012 PMC9810274

[37] Lefebvre S Bürglen L Frézal J Munnich A Melki J. The role of the SMN gene in proximal spinal muscular atrophy. Hum Mol Genet. 1998;7:1531–6. 9735373 10.1093/hmg/7.10.1531

